# Multifaceted effects of noisy galvanic vestibular stimulation on manual tracking behavior in Parkinson’s disease

**DOI:** 10.3389/fnsys.2015.00005

**Published:** 2015-02-02

**Authors:** Soojin Lee, Diana J. Kim, Daniel Svenkeson, Gabriel Parras, Meeko Mitsuko K. Oishi, Martin J. McKeown

**Affiliations:** ^1^Pacific Parkinson’s Research Centre, Department of Medicine, University of British ColumbiaVancouver, BC, Canada; ^2^Department of Electrical and Computer Engineering, University of New MexicoAlbuquerque, NM, USA

**Keywords:** Parkinson’s disease, vestibular system, GVS, manual tracking, discriminant analysis

## Abstract

Parkinson’s disease (PD) is a neurodegenerative movement disorder that is characterized clinically by slowness of movement, rigidity, tremor, postural instability, and often cognitive impairments. Recent studies have demonstrated altered cortico-basal ganglia rhythms in PD, which raises the possibility of a role for non-invasive stimulation therapies such as noisy galvanic vestibular stimulation (GVS). We applied noisy GVS to 12 mild-moderately affected PD subjects (Hoehn and Yahr 1.5–2.5) off medication while they performed a sinusoidal visuomotor joystick tracking task, which alternated between 2 task conditions depending on whether the displayed cursor position underestimated the actual error by 30% (‘Better’) or overestimated by 200% (‘Worse’). Either sham or subthreshold, noisy GVS (0.1–10 Hz, 1/f-type power spectrum) was applied in pseudorandom order. We used exploratory (linear discriminant analysis with bootstrapping) and confirmatory (robust multivariate linear regression) methods to determine if the presence of GVS significantly affected our ability to predict cursor position based on target variables. Variables related to displayed error were robustly seen to discriminate GVS in all subjects particularly in the Worse condition. If we considered higher frequency components of the cursor trajectory as “noise,” the signal-to-noise ratio of cursor trajectory was significantly increased during the GVS stimulation. The results suggest that noisy GVS influenced motor performance of the PD subjects, and we speculate that they were elicited through a combination of mechanisms: enhanced cingulate activity resulting in modulation of frontal midline theta rhythms, improved signal processing in neuromotor system via stochastic facilitation and/or enhanced “vigor” known to be deficient in PD subjects. Further work is required to determine if GVS has a selective effect on corrective submovements that could not be detected by the current analyses.

## INTRODUCTION

Motor symptoms in Parkinson’s disease (PD) characteristically manifest themselves as tremor, rigidity, akinesia/bradykinesia and postural instability. While levodopa is the gold standard treatment for PD, chronic use eventually leads to the long-term development of side effects, such as motor fluctuations, dyskinesias, and psychiatric disorders (Pontone et al., 2006; Weintraub et al., 2006). Surgical treatments, including deep brain stimulation targeted to subcortical nuclei, have provided effective therapeutic benefits, but are complex and invasive (Okun, 2012). With recent technological advances, numerous novel stimulatory techniques for PD treatment are presently being explored (Fuentes et al., 2009; Thevathasan et al., 2010; Samoudi et al., 2012; Faught and Tatum, 2013). Non-invasive brain stimulation techniques are currently a growing avenue of interest for PD and other neurological disorders due to their safety, tolerability and minimally invasive nature (Fregni and Pascual-Leone, 2007). Additionally, these methods, such as transcranial current brain stimulation (tCS), arguably influence solely the targeted site of stimulation, but also exert effects on associated brain connectivity patterns (Luft et al., 2014). Since PD is characterized by abnormally exaggerated beta synchronization throughout a basal ganglia (BG)-cortical network (Eusebio et al., 2009), non-invasive stimulatory approaches could potentially be used to modulate aberrant network dynamics (Fregni and Pascual-Leone, 2007).

A few studies have suggested that non-invasive stimulation of vestibular nerves via noisy galvanic vestibular stimulation (GVS) may improve motor deficits in PD (Yamamoto et al., 2005; Pan et al., 2008; Pal et al., 2009; Samoudi et al., 2012). Noisy GVS delivers currents with randomly varying amplitudes in time to vestibular afferents and subsequently influences resting state cortical electroencephalography (EEG) activity, suggesting that cortical-subcortical connections are also modulated by GVS (Kim et al., 2013). Akin to how tCS strengthens connectivity patterns in premotor, motor, and sensorimotor areas while subjects are engaged in a finger tapping task (Polanía et al., 2011), noisy GVS hypothetically is also able to influence functional BG-cortical motor networks depending on the brain state during stimulation. It is not fully established, however, whether noisy GVS improves motor performance. Yamamoto et al. (2005) measured trunk dynamics as well as reaction time in a Go/NoGo paradigm whereas Pan et al. (2008) measured wrist activity in akinetic PD patients. Effects of noisy GVS on postural and balance responses have also been measured in both humans and rat models (Pal et al., 2009; Samoudi et al., 2012), although none of these studies have directly investigated the effects of GVS on bradykinesia with respect to motor coordination and sensorimotor processing.

One potential way to rigorously assess the motoric effect of GVS is to utilize a visuomotor task, which is useful for understanding mechanisms that contribute to motor coordination with accuracy and stability (Ryu and Buchanan, 2012). Corrective movements and behavior are required in response to varying visual error feedback, which are important for maintaining effective perception-action or sensorimotor processing (Ryu and Buchanan, 2012). With respect to clinical significance, the ability to continually adapt one’s behavior to changing environmental or sensory stimuli is particularly relevant in PD as these patients demonstrate impaired switching between motor paradigms (Engel and Fries, 2010).

In the present study, we implemented a visuomotor tracking task and investigated the effect of noisy GVS on motor performance. Our visuomotor task required subjects to respond to visual error feedback that was, unbeknownst to the subjects, either minimized to 30% of the actual error, or amplified by 200% to create the appearance of ‘Better’ or ‘Worse’ motor performance, respectively. We used linear discriminant analysis (LDA; Duda et al., 2012) to identify parameters significantly influenced by GVS and to investigate if the effects of GVS are dependent on the task conditions. We then analyzed our data using a robust multivariate linear regression method (Filzmoser and Todorov, 2011) to test if tracking movement was affected by GVS. We show that subthreshold GVS resulted in robust changes in tracking, mostly related to increased sensitivity to perceived error.

## MATERIALS AND METHODS

### SUBJECTS

Twelve PD subjects (10 males, 2 females; mean age 61.4 ± 6.5 years; 11 right-handed, 1 left-handed) participated in the study. None of the participants had any reported vestibular or auditory disorders. All PD subjects were recruited from the Pacific Parkinson’s Research Centre (Vancouver, BC, Canada). PD subjects had mild to moderate disease severity (Hoehn and Yahr stages 1.5–2.5) with UPDRS (Unified Parkinson’s Disease Rating Scale) Part III motor scores at a mean of 22.3 ± 7.8 (**Table 1**). All PD subjects were tested in the off-medicated state after a 12-h overnight withdrawal from L-dopa medication. Other medications that some subjects were on included: amantadine, ramipril, and atorvastatin.

**Table 1:** PD subjects’ characteristics for behavior task.

Patient number	Age (yr)	Sex	Duration since diagnosis (yr)	UPDRS motor score	Hoehn and Yahr stage	Handedness
1	58	M	4	18	2	R
2	64	F	4	12	1.5	R
3	67	M	4	16	2	R
4	56	M	2.5	21	2	L
5	53	M	3	32	2.5	R
6	49	M	7.5	35	2	R
7	65	F	5	32	2	R
8	68	M	1.5	22	2	R
9	66	M	1	24	2	R
10	70	M	1	21	2	R
11	59	M	1.5	10	2	R
12	62	M	3.5	24	2	R

*UPDRS, unified Parkinson’s disease rating scale*.

### ETHICS STATEMENT

The study was approved by the University of British Columbia Clinical Research Ethics Board. All subjects gave written, informed consent prior to participation. Research was conducted according to the principles expressed in the Declaration of Helsinki.

### VISUOMOTOR TRACKING TASK

**FIGURE 1:**
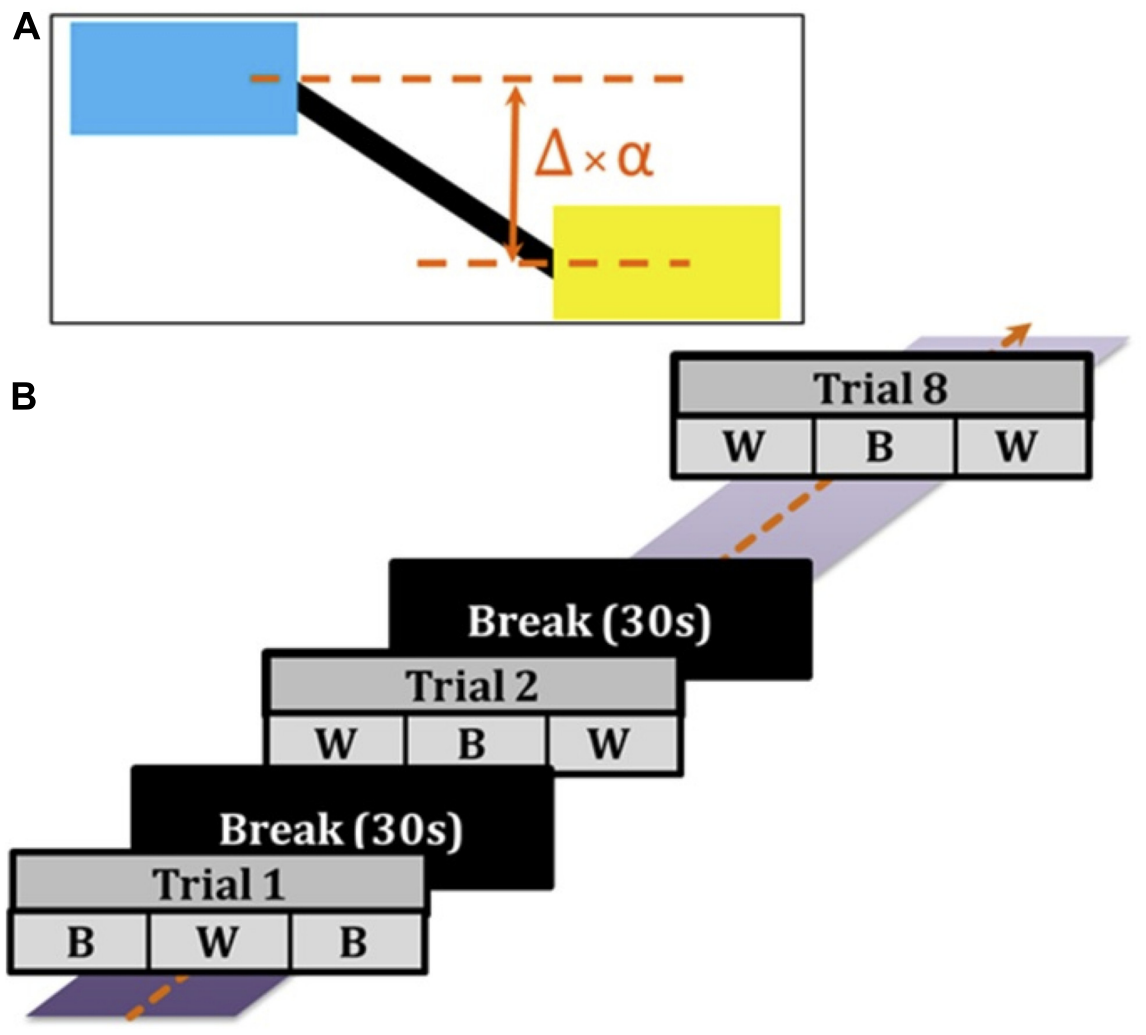
**Behavior task. (A)** Subjects faced a screen with a target (blue) that moved vertically up and down, and controlled a cursor (yellow) using a joystick. The error difference (Δ) between the actual positions of the target and cursor was amplified by a scaling factor (α): Δ ×**α** = displayed visual error feedback. In the ‘Better’ (B) condition, α was set to 0.3, and in the ‘Worse’ (W) condition, α was set to 2, such that it appeared that subjects performed better or worse respectively based on their visual error feedback. **(B)** Trials (90 s) alternated between B and W conditions (each condition 30 s). Each trial was followed by a break of 30 s until a culmination of eight trials total were completed for the experiment.

Subjects were comfortably seated 80 cm in front of a screen and performed a manual tracking task. On the screen, a target (blue) and cursor (yellow) connected by a black horizontal rod were displayed (**Figure 1**). The target box oscillated vertically up and down with the summation of two frequencies (0.06 and 0.1 Hz). Subjects controlled the cursor using a joystick with the objective of matching the horizontal position of the cursor to the target – i.e., to keep the horizontal black rod straight. The tracking error (Δ, difference between the actual positions of the target and cursor) was scaled by a factor (α) to determine the displayed position of the cursor: Δ × α = displayed visual error feedback. In the ‘Better’ (B) task condition, α was set to 0.3, and in the ‘Worse’ (W) task condition, α was set to 2, such that it artificially appeared to subjects that they performed better or worse, respectively, based on their scaled error feedback.

During the experiment, subjects performed a total of eight trials. Each trial (90 s) was comprised of three alternating blocks (30 s each) of B and W conditions – with Trial 1 ordered as B-W-B and Trial 2 ordered as W-B-W (**Figure 1**). During each trial, either a subthreshold *verum* current (90% of cutaneous sensory threshold) or *sham* current stimulation was delivered. Four trials contained *verum* GVS delivery whereas the other four trials contained *sham* stimulation. Subjects were unaware of either *verum* or *sham* stimulation since the order in which stimuli were delivered was pseudorandom, and the *verum* stimulation was imperceptible to the subject. Each trial was followed by a break (30 s) to preclude a hysteretic effect carrying over to the next trial. Before starting the experiment, subjects were allowed to practice tracking the target and using the joystick as needed in at least one practice trial. Practice trials were differently structured from the eight experiment trials described above. Due to technical details of the data capture system, the cursor position was irregularly sampled at ∼55 Hz. We then resampled the data at exactly 50 Hz using linear interpolation before further analyses.

### STIMULUS

Galvanic vestibular stimulation was delivered to subjects through carbon rubber electrodes (17 cm^2^) in a bilateral, bipolar fashion. For bilateral stimulation, an electrode was placed over the mastoid process behind each ear, and coated with Tac gel (Pharmaceutical Innovations, NJ, USA) to optimize conductivity and adhesiveness. The average impedance of the subjects was measured around 1 kΩ. Digital signals were generated on a computer using MATLAB and converted to analog signals via a NI USB-6221 BNC digital acquisition module (National Instruments, TX, USA). The analog command voltage signals were subsequently passed to a constant current stimulator (Model DS5, Digitimer, Hertfordshire, UK), which was connected to the stimulating electrodes.

**FIGURE 2:**
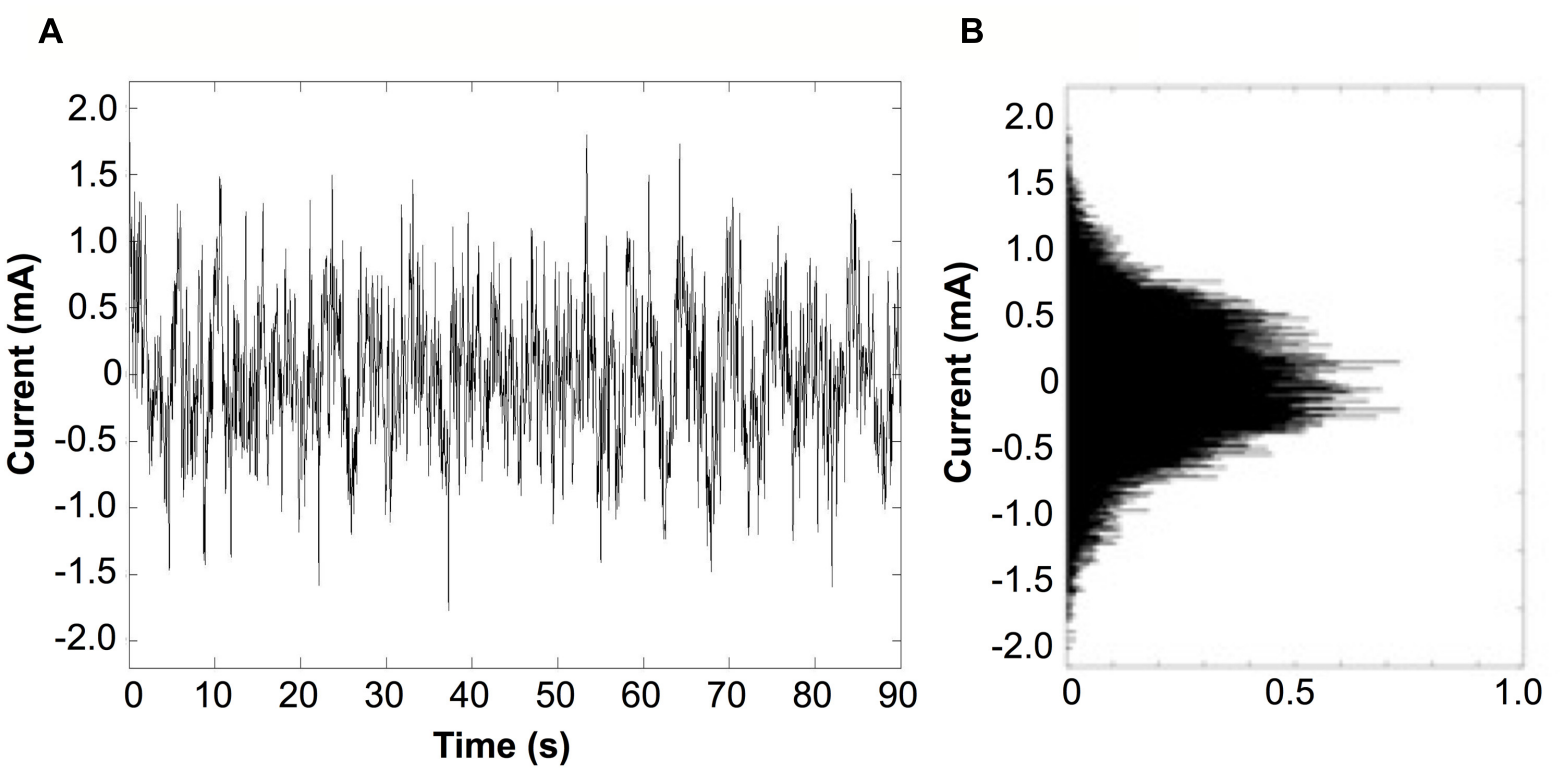
**Characteristics of the stimulus. (A)** Typical recording from a subject receiving a noisy stimulus applied for 90 s duration. The stimulus presented is at the highest current intensity (current level 6), which is set to 90% of the subject’s individual sensory threshold (RMS current value of 266 μA). **(B)** Probability density function of the stimulus current follows a Gaussian distribution.

Bipolar stimulation signals were zero-mean, linearly detrended, noisy currents with a 1/f-type power spectrum (pink noise) as previously applied to PD and healthy subjects (Soma et al., 2003; Yamamoto et al., 2005; Pan et al., 2008). The stimulation signal was generated between 0.1 and 10 Hz with a Gaussian probability density, with the command signal delivered to the constant-current amplifier at 60 Hz (**Figure 2**). The stimulus was applied at an imperceptible level to avoid effects by general arousal and/or voluntary selective attention, with the current level individually determined according to each subject’s cutaneous sensory threshold.

Since perception of GVS is inherently subjective, we utilized systematic procedures that have been previously used in determining subliminal current levels for both GVS and transcranial stimuli (Hummel et al., 2005; Utz et al., 2011; Wilkinson et al., 2012). Starting from a basal current level of 20 μA, noisy test stimuli were delivered for 20 s periods with gradual stepwise increases (20 μA) in current intensity until subjects perceived a mild, local tingling in the area of the stimulating electrodes. As performed previously, a threshold value was defined once subjects reported a tingling sensation (Utz et al., 2011; Wilkinson et al., 2012), which lasted for the duration of the test stimulus. The current level was then decreased each time by one level until sensation was no longer reported during delivery of test stimulus pulses, and increased by one step in current intensity to confirm threshold. Each delivery of a test stimulus was followed by a period of no stimulation for at least 30 s to preclude a hysteretic effect carrying over to the next test stimulus. Subjects were blind to the onset and duration of test stimuli, as well as the threshold-testing scheme. After completing the threshold test and throughout the experiment, stimuli were delivered at subthreshold intensity (190–900 μA), which is achieved at 90% of the determined cutaneous sensory threshold value.

### BEHAVIORAL DATA ANALYSIS

We employed both exploratory and hypothesis-driven analysis methods to analyze the behavioral data. We initially analyzed the data on a subject-by-subject basis as we were unclear whether or not there would be substantial intersubject variability to GVS response. LDA was first used to see if tracking behavior could be reliably discriminated depending upon whether GVS was applied or not. We derived a GVS linear discrimination function, g(X), to create maximum separation between means of the projected classes with minimum variance within each projected class:

(1) g(X)=X1w1+X2w2+...+X21w21+ω0=wtXt+ω0⁢

where ***X*** = [***X***_**1**_***X***_**2**_…***X***_**21**_] is a input data matrix in which each column represents an independent variable, ***w*** = [w_1_,w_2_,…, w_21_] ∈ℝ^21^ the weight vector containing linear coefficients of the variables in the data matrix ***X***, and ω_0_ the bias-weight. LDA was applied to the “Better” and “Worse” conditions separately.

For this exploratory part of the analysis, we included linear (first-order) and non-linear (second- and third-order) combinations of variables in the GVS discriminant function (**Table 2**). During the experiment, we varied the phase of the initial target trajectory not only between subjects but also between the trials to prevent the subjects from easily predicting upcoming target movement. Therefore, variables from X_1_ to X_9_ were included as nuisance variables in the LDA to account for the target differences.

**Table 2:** Variables in linear discriminant analysis (LDA) model.

Notation	Variables
X_1_,X_2_,X_3_	T(t),T(t)^2^,T(t)^3^
X_4_,X_5_,X_6_	V _T_(t),V _T_(t)^2^,V _T_(t)^3^
X_7_,X_8_,X_9_	A_T_(t),A_T_(t)^2^,A_T_(t)^3^
X_10_,X_11_,X_12_	D(t) -T(t),{D(t) -T(t)}^2^, {D(t) -T(t)}^3^
X_13_,X_14_,X_15_	V _D_(t) - V _T_(t),{V _D_(t) - V _T_(t)}^2^,{V _D_(t) - V _T_(t)}^3^
X_16_,X_17_,X_18_	D(t+Δt)-D(t),{D(t+Δt)-D(t)}^2^,{D(t+Δt)- D(t)}^3^
X_19_,X_20_,X_21_	V _D_(t + Δt) - V _D_(t),{V _D_(t + Δt) - V _D_(t)}^2^,{V _D_(t + Δt) -V _D_(t)}^3^

*T, target position; V _T_, target velocity; A_T_, target acceleration; D, displayed cursor position; V _D_, displayed cursor velocity; t, time index, and Δt, reaction delay of 0.5 s (Jordan et al., 1992)*.

To test for significance of the LDA results, we employed bootstrapping techniques. We permuted the GVS labels (on/off) and then re-computed the LDA function with the permuted data. This was repeated 1000 times. Any weight value from the original LDA function g(X) whose absolute value was greater than all the weights computed from the permuted data was considered to be significantly influenced by GVS.

In addition, a multivariate linear regression model was used to test the hypothesis that GVS had a significant effect on cursor position during tracking. As the traditional least squares regression may be sensitive to noisy and gross errors (Akkaya and Tiku, 2008), we chose a robust regression method to analyze our data (“robustfit” function in MATLAB). This method is known to be robust to outliers utilizing an iteratively reweighted scheme to deweight the influences of outliers. With cursor position as a response variable (Y _i_), the following regression model was proposed:

(2) $$Y_{i}=A_{i}\beta +\epsilon_{i}\,$$

where for each data point *i* we have the vector of independent variables ***A***_***i***_ = [A_i1_…,A_i5_], the vector of regression coefficients solved by a bisquare weighting function β, and the residual **ε**_***i***_ (assumed to be independent and identically distributed Gaussian). The selected independent variables are summarized in **Table 3** (note that A_1_, A_2_ and A_3_ are same as the variables X_1_, X_4_ and X_10_ in eq.1, respectively). The categorical variable of GVS was denoted with either 0 (GVSoff) or 1 (GVSon). We tested for significance of the coefficients under the null hypothesis that the coefficient estimates were equal to zero.

**Table 3:** Estimated coefficients in the robust regression model (eq.2) and the *p*-values.

Variables (*A*)	Coefficient estimates (β)	*p*-value
Target position (A_1_)	1.00	0.0000
Target velocity (A_2_)	-7.79e-02	0.0000
Displayed cursor position – target position (A_3_)	5.01e-01	0.0000
Cursor velocity – target velocity (A_4_)	-1.60e-02	0.0002
GVS (A_5_)	3.99e-05	0.0410

*R^2^ = 0.8811*.

For a signal-to-noise ratio (SNR) analysis, we utilized “snr” function in MATLAB to calculate SNR of cursor trajectories. This examines the fundamental frequencies of the tracking trajectory plus the next six harmonics, and assumes that any power in the spectrum than these peaks are “noise.”

## RESULTS

### RESULTS OF LDA IN WORSE CONDITION

**FIGURE 3:**
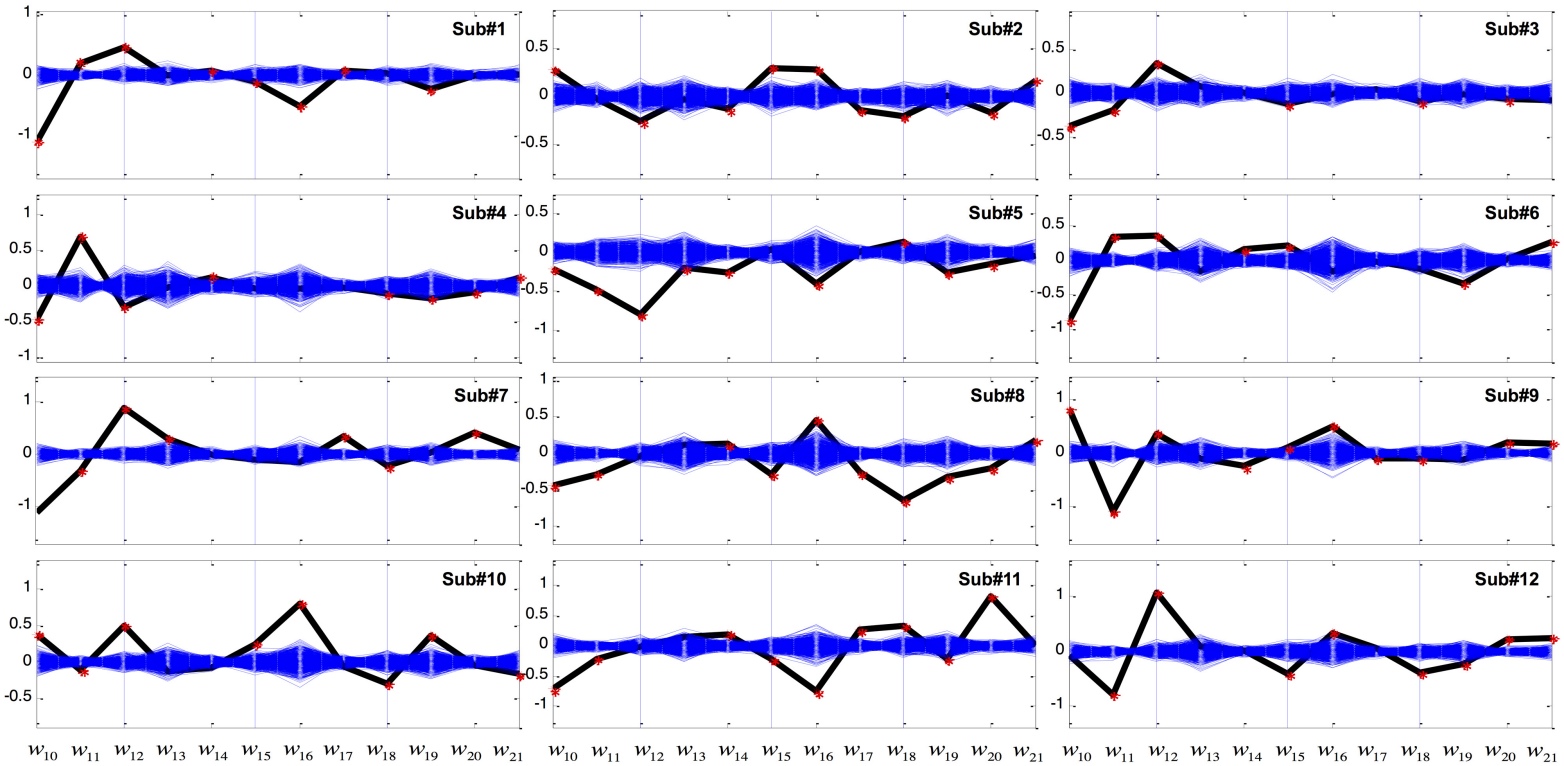
**Coefficients of the variables of the linear discriminant function in the Worse condition.** The *x*-axis represents variables from X_10_ to X_21_ in **Table 2** while the *y*-axis represents weight (w) value. The computed coefficients are depicted as black for the GVS discriminant function and blue for bootstrapping. Red asterisks denote coefficients that are outside the 95% confidence interval of bootstrapping.

Coefficients of GVS discriminant function (eq.1) were calculated for each subject and are plotted as black lines in **Figure 3**. For clarity, nuisance variables related to absolute target position (i.e., X_1_–X_9_) are not shown. The 1000 sets of linear coefficients generated from the bootstrapping are depicted as blue lines. In most subjects, the coefficients w_10_, w_11_, and w_12_ of g(X; representing linear and higher powers of the perceived error between the target and the displayed cursor position) were robustly modulated by GVS. In addition, displayed cursor velocity (w_16_ or w_17_) and acceleration (w_19_, w_20_, or w_21_) were also found to be significantly affected by GVS across subjects.

### RESULTS OF LDA IN BETTER CONDITION

**FIGURE 4:**
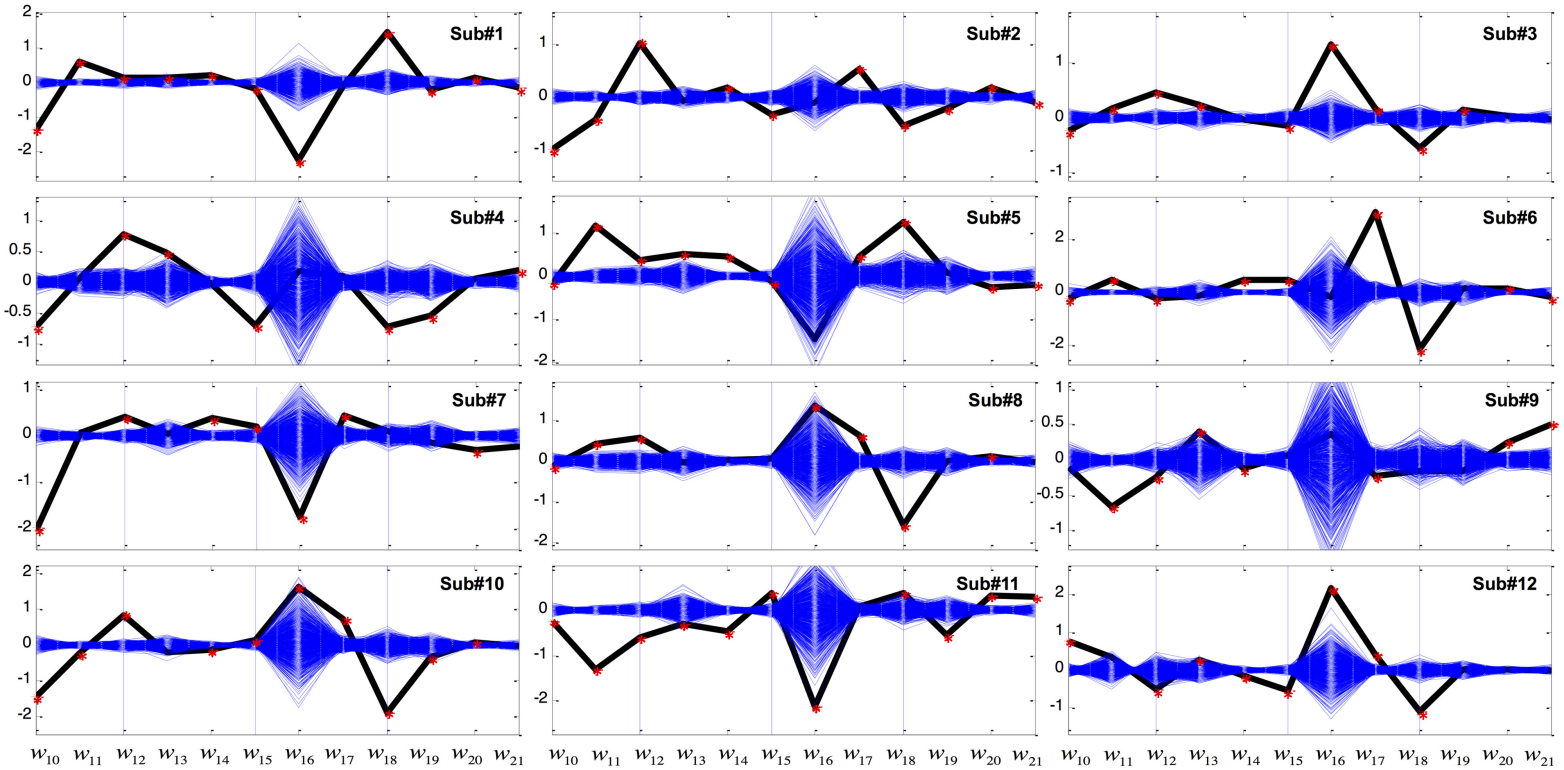
**Coefficients of the variables of the linear discriminant function in the Better condition.** The *x*-axis represents variables from X_10_ to X_21_ in **Table 2** while the *y*-axis represents weight (w) value. The computed coefficients are depicted as black for the GVS discriminant function and blue for bootstrapping. Red asterisks denote coefficients that are outside the 95% confidence interval of bootstrapping.

**Figure 4** shows the LDA results in the better condition. As before, coefficients w_10_, w_11_, and w_12_ were significant among all the subjects. In addition, 10 out of 12 subjects showed significant w_18_ weightings. Other coefficients were not robustly seen in all subjects. For example, unlike the LDA results of the Worse condition, displayed cursor acceleration (w_19_, w_20_, or w_21_) was no longer significantly influenced by GVS in the Better condition.

### RESULTS OF ROBUST REGRESSION MODEL

**Table 3** is the coefficient estimates of the variables of the multivariate regression model (eq.2) and their *p*-values. The computed *R*^2^ of the regression model was 0.8811. GVS was significantly associated with cursor position across all subjects (*p* < 0.05).

### EFFECT OF GVS ON CURSOR OVERSHOOTING

**FIGURE 5:**
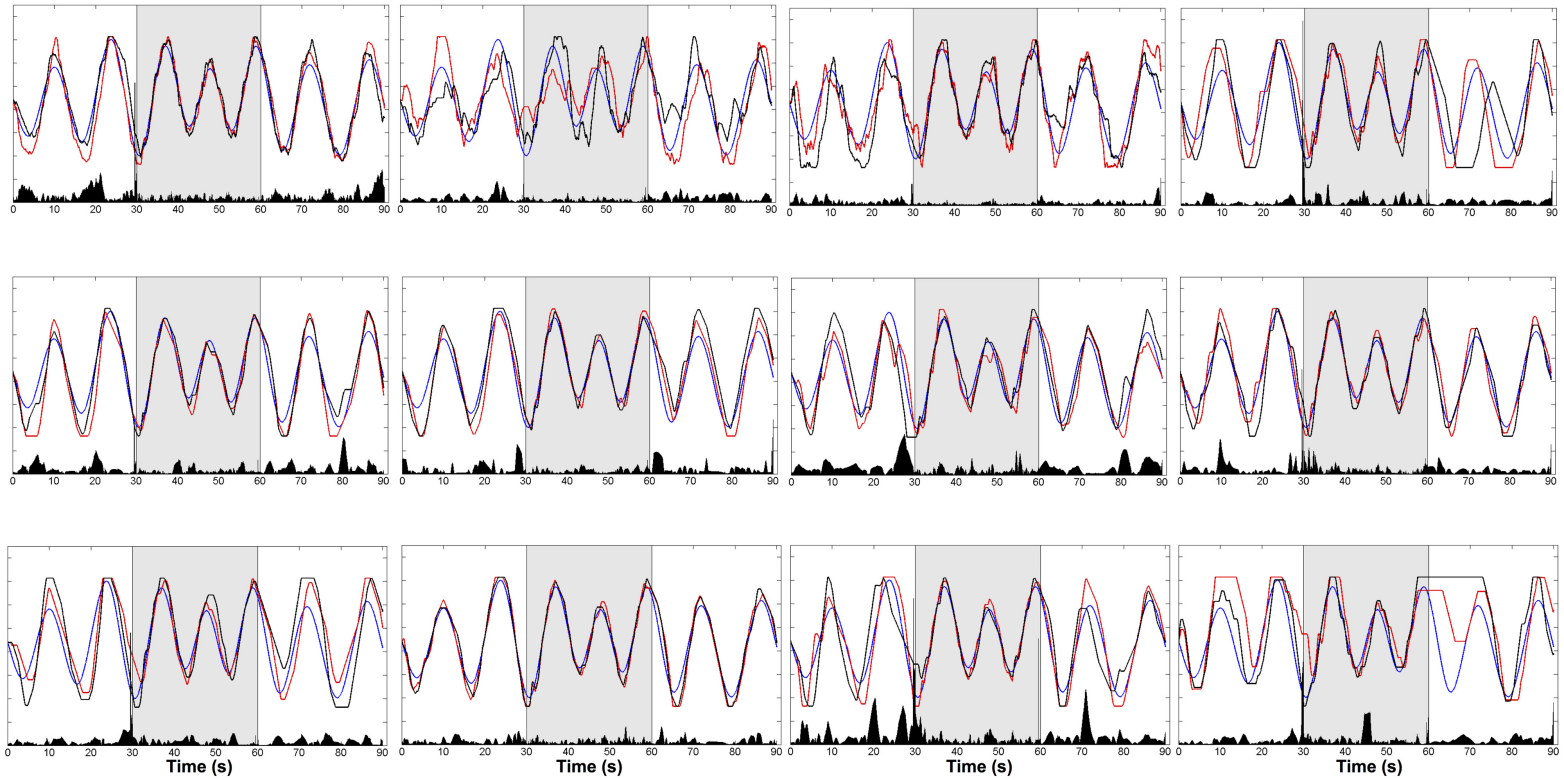
**Trajectories of target (blue) and cursor (GVSon: red, GVSoff: black) and Δ**g** (black bar in the bottom).** Δg was computed by subtracting the linear discriminant function values of trial 7 (GVSoff) from trial 1 (GVSon).The trials alternated between W-B-W conditions (each condition 30 s).

In order to get an intuitive interpretation of GVS effects, we calculated the GVS discriminant function values (eq.1) for each subject. We used data from trials 1 and 7 for the calculation as these two trials had identical phases of the trajectories, with a difference in whether or not GVS was delivered (GVSon for trial 1). Then, Δg was computed by subtracting the function values of trial 7 from trial 1. By plotting Δg, we could not only locate GVS effects on the cursor trajectory but also directly make visual comparison of the cursor movement in the identified location. **Figure 5** shows target trajectory, cursor trajectory and Δg for each subject.

The effect of GVS was greatest near sinusoidal peaks. This trend was found in most of the subjects regardless of how well the subjects tracked the target. For instance, subject 5 tracked the target relatively better compared to the other subjects, and Δg was significant around at 5, 20, 65, and 80 s. Subjects 11 and 12 performed the tracking task poorly, but the GVS effects still appeared near sinusoidal peaks.

**FIGURE 6:**
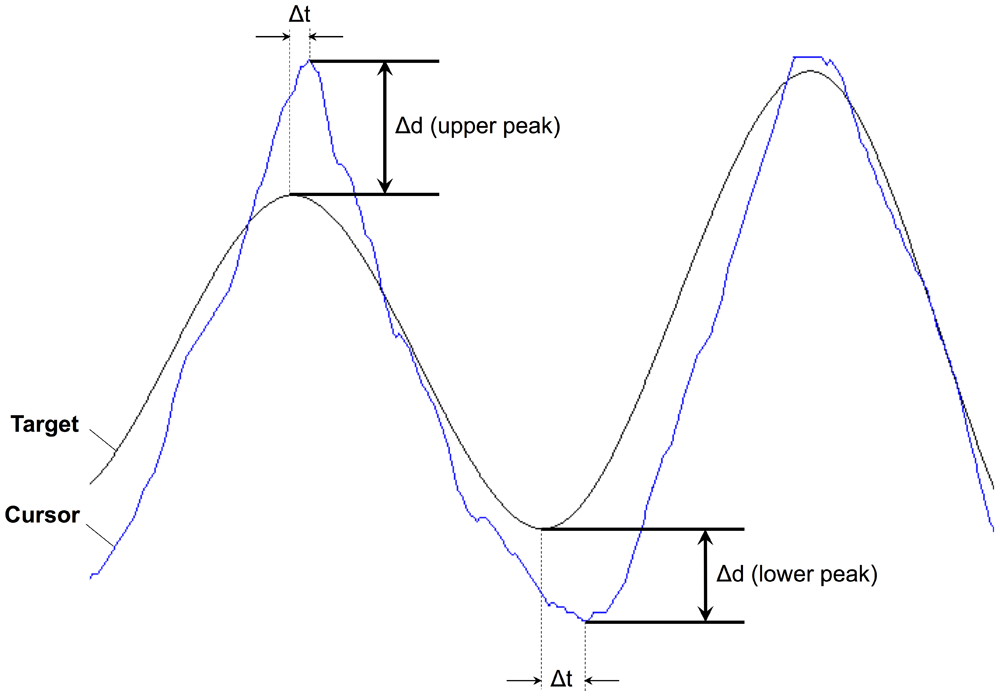
**Representative example of cursor overshooting on upper and lower peaks from Subject 1 Cursor overshooting (Δd) was calculated as cursor position – target position.** Δt represents time difference between peaks in cursor and target trajectories.

One of the noticeable features on the peaks is a degree of overshooting of cursor trajectories. To assess a possible relationship to GVS stimulation, we compared the difference between the cursor position and the target on the peaks. **Figure 6** shows a representative example of cursor overshooting near sinusoidal peaks in target. The peaks in cursor appeared with some lagged time (Δt). The amplitude of the target peaks was subtracted from the cursor peaks, and the difference (Δd) was defined as cursor overshooting. Cursor peak was defined when the cursor position was at its max/min point. Cursor overshooting was calculated for all trials and subjects, then averaged depending on the task conditions and presence of GVS stimulation as shown in **Table 4**. The *p*-value was calculated from ANOVA of the means between GVSon and GVSoff (i.e., a single, two-level factor).

**Table 4:** Means of cursor overshooting on sinusoidal peaks and ANOVA results.

	Lower peak			Upper peak		
	GVSon	GVSoff	*p*-value	GVSon	GVSoff	*p*-value
Worse	-0.0517	-0.0714	0.0036	0.0695	0.0784	0.22
Better	-0.0946	-0.0451	0.0038	0.0890	0.0690	0.14

In Worse condition, the subjects tended to overshoot significantly less on the lower peaks while stimulated by GVS. On the upper peaks, the mean overshooting of GVSon was also smaller than GVSoff, but the difference was not significant. In Better condition, however, there was an increasing tendency for cursor overshooting with stimulation.

### EFFECT OF GVS ON SNR OF CURSOR TRAJECTORY

**FIGURE 7:**
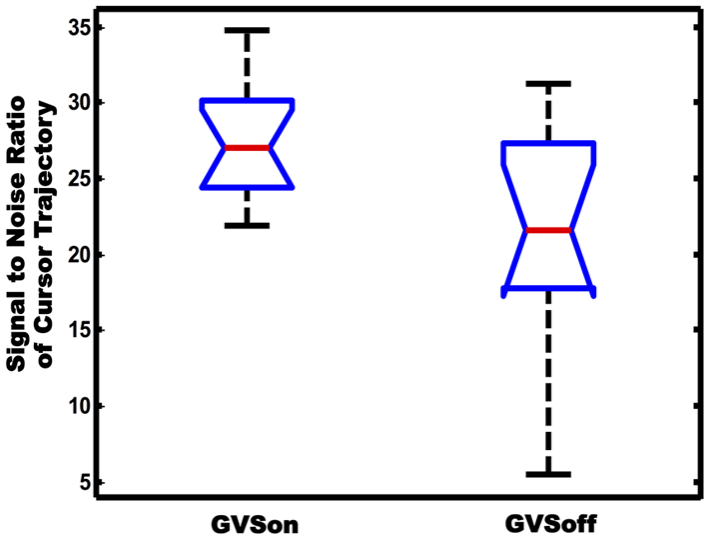
**Comparison of SNR of cursor trajectories between GVSon and GVSoff conditions**.

Movement variability is another important feature to characterize the tracking performance. Particularly, in goal-directed behavior, the variability originates from collateral movement to the main goal of a task. In this sense, the cursor trajectories in our tracking test can be seen to a combination of two components. One is the primary movement whose form is similar to the target trajectory, and the other is submovement that may appear as noise superimposed on the primary movement. In order to investigate if GVS had affected movement variability of the subjects, we calculated SNR of cursor trajectories and compared differences in between GVSon and GVSoff conditions. As shown in **Figure 7**, the mean SNR of 12 PD subjects was 27.6 when GVS was applied, which was significantly greater than 21.3 in GVSoff condition (*p* < 0.05).

## DISCUSSION

Our results demonstrate that noisy GVS robustly influences motor tracking performance in PD patients off dopaminergic medication. Motor improvements are consistent with results previously reported in hemiparkinsonian rats (Samoudi et al., 2012) whereby GVS with a 1/f power density improved rod performance. Previously, we demonstrated that noisy GVS has the ability to modulate synchronization of broadband EEG oscillations in healthy subjects (Kim et al., 2013). Our recordings of EEG rhythms were observed at resting-state, suggesting that noisy GVS was able to modulate cortical activity and presumably connected subcortical-cortical projections. In this study, we observed a functional effect of GVS on sensorimotor processing and motor performance in a visuomotor task, suggesting that noisy vestibular stimulation modulates motor networks in PD subjects.

Our results seem to indicate that noisy GVS affects the sensitivity of motor responses (in this case, joystick-controlled cursor position) to visualized error (displayed cursor position – target position). We do not believe that our observed results are the consequence of an attentional or general arousal effect, such as through activation of the reticular activating system. The imperceptible nature of our stimulus, which subjects were not aware of throughout the experiment trials, precludes this issue which is present with other forms of minimally invasive stimulation methods (Fuentes et al., 2009).

Depending on the stimulus parameters (i.e., current intensity, frequency, signal shape), GVS is known to induce a broad range of effects, including eye movements, postural control and movements (Fitzpatrick and Day, 2004). Therefore, one interpretation of our results may include the confounding effects of nystagmus and/or ocular torsion through activation of the vestibulo-ocular reflex (VOR; Zink et al., 1998). Since subjects rely on visual error feedback, ocular torsion would potentially hamper the perceived error feedback through a subjective tilt in the visual perceptual field (Zink et al., 1998). However, we note that our stimulus levels were weak, subthreshold currents with the highest current delivered at around 140 ± 113 μA, whereas the preferred GVS current intensities for inducing ocular torsion and subsequent perceptual tilts through GVS are much higher at around 1–3 mA (Zink et al., 1998). Therefore, we presume that our subthreshold stimulus was not strong enough to notably induce confounding visual effects and corollary perceptual changes in our experiment.

Noisy GVS is known to modulate EEG spectral power. Wilkinson et al. (2012) have demonstrated that noisy GVS is able to modulate the EEG spectral power during a face processing task. Our previous study has demonstrated that noisy GVS is able to modulate the EEG synchrony patterns in healthy subjects (Kim et al., 2013). Altogether, these findings combined with our present results suggest that noisy GVS is able to modulate oscillatory activity in resting and task-related networks, which involve sensorimotor processing in our particular study.

The motoric effects of GVS may be related to modulation of oscillations related to integration of information and error-processing. Since perceived error (i.e., the error between the target and the displayed cursor position) was robustly detected by the LDA analysis, fronto–midline (FM) theta may be a candidate oscillation to be modulated by GVS in PD subjects. FM-theta shows an increased amplitude during tasks requiring concentration (Mitchell et al., 2008), which is related to error-related negativity (ERN), an event-related potential seen after errors are made. FM-theta may represent a universal mechanism for action monitoring with the midcingulate cortex acting as hub for the integration of information (Cavanagh et al., 2012). Thus, our results suggest that GVS may regulate FM-theta activity in PD subjects.

The increased SNR shown in **Figure 7** suggests that application of noisy GVS may have increased synchronization in neuromotor system via stochastic facilitation. Stochastic facilitation is a term to describe phenomena where stochastic biological noise elicits functional benefits in a non-linear system such as the nervous system (McDonnell and Ward, 2011). Several studies have reported that a presence of additive noise allows a weak input signal to be better detected, resulting in an increase in SNR in EEG (Galambos and Makeig, 1992; Srebro and Malladi, 1999; Elias et al., 2003; Kitajo et al., 2007; Keita et al., 2008; Ward et al., 2010; Doren et al., 2014) and sensorimotor performance (Ignacio et al., 2012). These findings suggest that noisy GVS input may also be able to modulate detection and transmission of the sensorimotor system via stochastic facilitation, resulting in an increase in synchronization of the neuromotor system. However, a further investigation is required to elucidate whether the synchronization is limited to cortical areas or if it could give rise to corticomuscular synchronization (Ignacio et al., 2012).

We further speculate that our results may be at least partly explained by modulation of cortico-BG rhythms involved in sensorimotor processing. Growing observations suggest a concept that the BG regulates action motivation or response ‘vigor’ (Niv et al., 2007; Salamone et al., 2009) as well as the speed and size of movement (Spraker et al., 2007; Thobois et al., 2007). Deficient scaling of the initial burst of earliest agonist muscle activity (EMG) to meet the demands of a motor task is frequently observed in clinical disorders of the BG, such as PD. The link between motivation and movement gain may be universally weakened in Parkinsonian subjects (Ballanger et al., 2006; Thobois et al., 2007). We thus speculate that GVS may also correct deficient vigor caused by BG dysfunction through modulation of pathological brain rhythms.

We note that we used a single noisy stimulus for all subjects. However, the results shown in **Figure 3** also emphasize the importance of looking at patient-specific stimuli. For instance, the coefficients regarding the difference between cursor and target velocities (w_13_, w_14_, and w_15_) were found to be significant in some subjects, but were indistinguishable from bootstrapping for the rest subjects.

Finally, we note that GVS had fewer effects in the Better condition compared to the Worse condition. Presumably, subjects would have made fewer corrective movements in the former condition. This raises the possibility that GVS may also depend upon the number and form of corrective submovements. As submovements were not captured by the global LDA and multivariate regression methods used here, this warrants further investigation.

## Conflict of Interest Statement

The authors declare that the research was conducted in the absence of any commercial or financial relationships that could be construed as a potential conflict of interest.
